# Underreporting of hepatitis A in non-endemic countries: a systematic review and meta-analysis

**DOI:** 10.1186/s12879-016-1636-6

**Published:** 2016-06-13

**Authors:** Rachel D. Savage, Laura C. Rosella, Kevin A. Brown, Kamran Khan, Natasha S. Crowcroft

**Affiliations:** Dalla Lana School of Public Health, University of Toronto, 155 College St, 6th Floor, Toronto, Ontario M5T 3M7 Canada; Public Health Ontario, 480 University Ave, Suite 300, Toronto, Ontario M5G 1V2 Canada; Institute for Clinical Evaluative Sciences, Veterans Hill Trail, 2075 Bayview Avenue G1 06, Toronto, Ontario M4N 3M5 Canada; St. Michael’s Hospital, 30 Bond St, Toronto, Ontario M5B 1W8 Canada; Department of Medicine, University of Toronto, Medical Sciences Building, 1 King’s College Cir #3172, Toronto, Ontario M5S 1A8 Canada; Department of Health Policy, Management and Evaluation, University of Toronto, 155 College St, 4th Floor, Toronto, Ontario M5T 3M7 Canada; Department of Laboratory Medicine and Pathobiology, University of Toronto, Medical Sciences Building, 6th Floor, 1 King’s College Cir, Toronto, Ontario M5S 1A8 Canada

**Keywords:** Hepatitis A, Population surveillance, Disease notification, Underreporting

## Abstract

**Background:**

Information on reporting completeness of passive surveillance systems can improve the quality of and public health response to surveillance data and better inform public health planning. As a result, we systematically reviewed available literature on reporting completeness of hepatitis A in non-endemic countries.

**Methods:**

We searched Medline, EMBASE and grey literature sources, restricting to studies published in English between 1997 and 21 May 2015. Primary studies on hepatitis A surveillance and underreporting in non-endemic countries were included, and assessed for risk of bias. A pooled proportion of reporting completeness was estimated using a DerSimonian-Laird random-effects model.

**Results:**

Diagnosed hepatitis A cases identified through positive laboratory tests, physician visits, and inpatient hospital discharges were underreported to public health in all eight included studies. Reporting completeness ranged from 4 to 97 % (pooled proportion 59 %, 95 % confidence interval = 32 %, 84 %). Substantial heterogeneity was observed, which may be explained by differences in the referent data sources used to identify diagnosed cases and in case reporting mechanisms and/or staffing infrastructure. Completeness was improved in settings where case reporting was automated or where dedicated staff had clear reporting responsibilities.

**Conclusions:**

Future studies that evaluate reporting completeness should describe the context, components, and operations of the surveillance system being evaluated in order to identify modifiable characteristics that improve system sensitivity and utility. Additionally, reporting completeness should be assessed across high risk groups to inform equitable allocation of public health resources and evaluate the effectiveness of targeted interventions.

**Electronic supplementary material:**

The online version of this article (doi:10.1186/s12879-016-1636-6) contains supplementary material, which is available to authorized users.

## Background

Hepatitis A is a disease caused by infection with hepatitis A virus (HAV), which is most often transmitted through the faecal-oral route. In young children, infection with HAV is typically asymptomatic, while in older children and adults, infection leads to jaundice in approximately 70 % of cases [1]. The disease is usually self-limiting; however, approximately one in four cases are hospitalized, the proportion increasing with age [2, 3]. In low-income countries, where the disease is considered endemic (i.e. the prevalence of anti-HAV antibodies in the population is ≥ 90 % by age 10), most infections occur before the age of 5 years, when infections are asymptomatic and as a result, there are few susceptible adolescents or adults and few symptomatic infections [1]. By contrast, in high-income, non-endemic countries, the prevalence of anti-HAV antibody is very low (<50 % are immune by age 30) [1] owing to improved sanitary conditions, along with introduction of an effective vaccine in the mid 1990’s. In Canada, reported rates have declined from 10.6 cases per 100,000 population in 1991 to 0.9 cases per 100,000 population in 2008 [4]. Similar trends have been observed in the United States (US) [3, 5]. The risk of infection is low in these countries, despite a large population of susceptible adults, because the high standards of living have reduced the effective reproduction number below 1, meaning a very low risk of exposure as there is little circulation of the virus. Despite this, hepatitis A remains of public health importance. In 2012 and 2013, outbreaks affecting more than 250 individuals were reported across Europe, Canada and the US as a consequence of travel to endemic regions, as well as importation of contaminated frozen fruit from endemic regions [6–8]. Susceptible populations also remain at high risk for exposure, including travelers to endemic regions, men who have sex with men and injection drug users [1].

Hepatitis A cases are typically diagnosed by physicians on the basis of clinical and epidemiological features, as well as serological testing to detect immunoglobulin M (IgM) antibody to HAV [5]. The majority of World Health Organization (WHO) Member States (71 %, 90/126) reported having a national surveillance system for acute hepatitis A infection in a 2012 survey; however, only 47 % of low-income countries responded to the survey (compared to 80 % of high-income countries) making it challenging to understand the true landscape of hepatitis A surveillance globally [9]. In most high-income countries, hepatitis A is a notifiable disease. While no universal case definition exists, in the US, cases reported to public health are classified as confirmed if they have acute illness with discrete onset of symptoms, and jaundice or elevated serum aminotransferase levels, and are laboratory-confirmed (IgM antibody to HAV (anti-HAV positive)), or they meet the clinical case definition and have an epidemiologic link with a person who has laboratory-confirmed hepatitis A [10]. Epidemiological surveillance is conducted passively through physician and/or laboratory reporting in order to detect and control outbreaks, as well as to guide and evaluate public health interventions.

A recognized limitation of these data are their sensitivity (i.e. data underestimate the total number of cases in the population). System sensitivity is a function of both case ascertainment and disease reporting. Under-ascertainment refers to cases not captured in a surveillance system due to fact that health care was not sought; this can occur if cases are asymptomatic, if they do not visit a care provider due to mild symptoms or other reasons, or if their care provider does not test for the disease [11, 12]. An estimated 15 % of hepatitis A cases are asymptomatic (although this proportion can be as high as 70 % in children <6 years [13]) and 12 % of symptomatic patients are estimated to not seek care [14]. Underreporting, by contrast, refers to cases that are diagnosed but are not reported to the appropriate public health authorities; this can result from a lack of knowledge that the disease is notifiable and/or understanding of how to report the disease, as well as errors in the reporting system/mechanism [11, 12].

While not all cases of hepatitis A need to be captured to achieve maximum control levels (as not all secondary cases need to be prevented to bring the number of infections produced on average per case to <1, which is required for control), a modelling study examining the impact of reporting delays found that hepatitis A outbreak control would not possible with underreporting >29 % [15]. As a result, ongoing evaluation and improvement of surveillance systems is necessary to optimize control efforts. Similarly, completeness of disease ascertainment and reporting should be assessed and corrected for when estimating the burden of disease from surveillance data, as illustrated by recent infectious disease burden studies in Canada and Europe [16, 17]. Adjustment is particularly important across key epidemiological characteristics such as age, sex and risk status, as completeness of data have been shown to be related to these characteristics [18, 19]. This information can, in turn, better inform public health planning, priority setting and equitable distribution of limited resources.

While the literature on reporting completeness for notifiable diseases has been synthesized overall and for broad disease groups in the US and the United Kingdom [12, 20], no such reviews exist for hepatitis A. Disease-specific data are important as the degree of underreporting has been shown to vary substantially by disease [12, 20]. As a result, the objective of this study was to determine the proportion of diagnosed hepatitis A cases that were reported to public health bodies or agencies in non-endemic countries. A secondary objective was to synthesize and critically appraise the methods used to estimate underreporting to provide recommendations for future studies.

## Methods

We followed guidelines from the Preferred Reporting Items for Systematic Reviews and Meta-Analyses (PRISMA) 2009 statement [21], which is available in Additional file 1.

### Search and study selection

Medline and EMBASE databases were searched on 21st May 2015. A sensitive strategy comprising two searches was designed in consultation with two public health librarians. In search one, mapped subject headings and keywords for hepatitis A were joined with terms for disease notification, surveillance and underreporting. In search two, search terms for hepatitis A were joined with terms for incidence, prevalence and epidemiology, as well as travel, non- or low-endemic countries, and country names. World regions with estimated ‘very low’ endemicity, defined as <50 % of the population immune by age 30, were classified as non-endemic [22]. The Medline search strategy is shown in Table 1. The scope was limited to non-endemic countries to minimize heterogeneity in factors known to affect disease surveillance and reporting, including disease exposure, surveillance and health care systems, socioeconomic development, and availability of treatments [11]. The search was restricted to English articles published after 1 January 1997 to limit the review to the time period in which the hepatitis A vaccine was introduced (1996 in Canada; 1995 and 1996 in the US) [4, 23]. Commentaries, editorials, letters, news reports and case reports were excluded. Reference lists of all studies included for full-text review were manually searched for relevant studies. Additionally, a snowball search was performed for all included articles using the ‘find all related’ function in PubMed to retrieve studies on underreporting of multiple infectious diseases that did not map ‘hepatitis A’ as a subject heading or keyword but nonetheless may have included it as a disease of interest.

**Table 1:** Example Medline search strategy to systematically retrieve literature on hepatitis A underreporting

Search 1^a^	Search Terms
Disease notification, surveillance and underreporting	Mandatory Reporting/ OR Disease Notification/ OR Contact Tracing/ OR exp Population Surveillance/ OR Public Health Informatics/ OR exp Data Collection/ OR Disclosure/ OR exp Informatics/ OR underreport$.mp. OR under-report$.mp. OR (under adj1 report$).mp. OR surveillance.mp. OR reporting.mp. OR ((reported OR true OR estimate$) adj2 (incidence OR prevalence)).mp. OR undetect$.mp. OR capture-recapture.mp. AND
Hepatitis A	Hepatitis A/ OR Hepatitis A Virus, Human/ OR Hepatitis A Antibodies/ OR “hepatitis a”.mp.
Search 2^a^	Search Terms
Incidence, prevalence and epidemiology	Incidence/ OR Prevalence/ OR Epidemiology/ OR Statistics & Numerical Data.fs. OR Epidemiology.fs. OR Epidemiologic Measurements/ OR Statistics as Topic/ OR Data Interpretation, Statistical/ OR incidence.mp. OR prevalence.mp. OR epidemiolog$.mp. AND
Hepatitis A	Hepatitis A/ OR Hepatitis A Virus, Human/ OR Hepatitis A Antibodies/ OR “hepatitis a”.mp. AND
Travel, non- or low-endemic countries	Travel/ OR Travel Medicine/ OR Developed Countries/ OR exp Australia/ OR exp North America/ OR New Zealand/ OR Andorra/ OR Austria/ OR Belgium/ OR Finland/ OR exp France/ OR exp Germany/ OR Gibraltar/ OR exp Great Britain/ OR Greece/ OR Iceland/ OR Ireland/ OR exp Italy/ OR Liechtenstein/ OR Luxembourg/ OR Monaco/ OR Netherlands/ OR Portugal/ OR San Marino/ OR exp Scandinavia/ OR Spain/ OR Switzerland/ OR Japan/ OR Singapore/ OR Hong Kong/ OR (non-endemic$ OR nonendemic$ OR (non adj1 endemic$) OR low-endemic$ OR (low adj1 endemic$) OR travel$ OR developed countr$ OR Australia OR North America OR Canada OR United States OR New Zealand OR Western Europe OR Andorra OR Austria OR Belgium OR Finland OR France OR Germany OR Gibraltar OR Great Britain OR England OR Scotland OR Ireland OR United Kingdom OR Wales OR Greece OR Iceland OR Italy OR Liechtenstein OR Luxembourg OR Monaco OR Netherlands OR Portugal OR San Marino OR Scandinavia OR Spain OR Switzerland OR Cyprus OR Denmark OR Norway OR Sweden OR Greenland OR Japan OR Singapore OR Hong Kong).mp.

^a^Searches 1 and 2 were combined using the Boolean operator ‘or’. *exp* explode – includes all narrower/more specific subheadings in the search, $, allows for different endings to a word to be searched; *mp* multi-purpose - searches in the title and abstract as well as the subject heading, *adj* adjacent, *fs* floating subheading – facilitates a broader search (floats over all indexed subject headings)

Grey literature sources, including websites of the World Health Organization (WHO), United States Centers for Disease Control and Prevention (US CDC), Public Health Agency of Canada (PHAC), European Centre for Disease Prevention and Control (ECDC) and EUROHEP.NET were searched using keywords; these searches were restricted to English and to 2010–2013. Abstract books from the European Scientific Conference on Applied Infectious Disease Epidemiology (ESCAIDE), the International Conference on Emerging Infectious Diseases (ICEID), and ID Week, held between 2009 and 2013, were also searched.

The primary reviewer (R.D.S.) performed a preliminary review of article titles and abstracts to exclude irrelevant studies on non-viral hepatitis, chronic hepatitis, liver cancer, non-A hepatitis, laboratory detection methods for hepatitis, and surveillance in non-humans (e.g. animals, food, water). The remaining article titles and abstracts were independently reviewed by two reviewers (R.D.S. and K.A.B.); primary studies on hepatitis A surveillance and underreporting in non-endemic countries were considered relevant for full-text review. Mathematical modelling papers on hepatitis A were also reviewed in full-text as a validation measure to ensure all relevant primary studies were captured in the search, as these models often account for underreporting. Discordant views on eligibility were discussed to reach consensus. Inter-rater reliability between the two reviewers was assessed using the kappa statistic, where >0.75 indicated excellent agreement, 0.40–0.75 intermediate to good agreement, and <0.40 poor agreement [24].

Full-text review was then performed independently by the two reviewers (R.D.S. and K.A.B.); studies were eligible for inclusion if they assessed underreporting by comparing hepatitis A disease reports received by public health with one or more independently retrieved data source(s) of diagnosed cases as the reference standard (e.g. IgM anti-HAV positive laboratory test results). Studies whose aims were to exclusively identify the source of case reports (i.e. evaluate the proportion of cases that were reported to public health by physicians, laboratories, etc.) were excluded as they did not make comparisons with a reference standard. Studies also needed to provide a quantitative measure of underreporting or provide data to calculate the degree of underreporting for hepatitis A. Reasons for exclusion were recorded, and as before, discordant views on eligibility were discussed to reach consensus and inter-rater reliability was assessed.

### Data collection

The primary reviewer (R.D.S.) extracted data on the last name of the first author, year of publication, study setting, the passive surveillance system used to report hepatitis A cases, the data source of diagnosed cases used to measure underreporting, the proportion of diagnosed cases reported to public health overall and by age, sex and/or risk status if provided, and the study’s sample size using a structured data extraction form.

### Risk of bias

The methodological quality of included studies was assessed independently by two reviewers (R.D.S. and K.A.B.) using Public Health Ontario’s meta-tool for quality assessment of public health evidence (Table 2) [25]. The validated tool uses a component or checklist approach to facilitate the assessment of four domains: relevancy, reliability, validity and applicability. The tool integrates adapted study-design specific critical appraisal tools and reporting guidelines, such as PRISMA [21], CONSORT [26] and CASP [27], to assess the study’s validity; however, finding that none of these tools aligned with the study designs included in this review, a checklist was created based on two existing reviews of reporting, as well as the US CDC’s updated guidelines for evaluating public health surveillance systems [12, 28, 29]. If the study methodology was inappropriate, biased (e.g. failed to validate diagnosis in the data source of diagnosed cases), or unclear (e.g. failed to describe diagnostic or data linkage methods), the study was assigned a high risk of bias. If the methodology was appropriate, clearly defined, and bias-free, the study was assigned a low risk of bias. If the study presented findings objectively with justified conclusions, but the methods were unclear or potentially biased, the study was rated as moderate risk. Inter-rater reliability was assessed using the kappa statistic.

**Table 2:** Adapted meta-tool for quality assessment of public health evidence (Meta QAT) from Public Health Ontario [25]

Item	Criteria	Assessment
Validity	a) Are findings presented objectively? • Clear rationale and justification • Findings presented and discussed within appropriate context • Similar to existing literature and if not, reasons explained • Conflict of interest statement	Yes No Unclear N/A
	b) Are the authors conclusions justified? • Transparency • Results consistent with those described in discussion • Conclusions based on empirical findings	Yes No Unclear N/A
Reliability	a) Is the research methodology clearly described? • Study population and surveillance system described • Can identify the research design • Methods carried out in a rigorous and transparent manner	Yes No Unclear N/A
	b) Is methodology appropriate for the scope of research?^a^ • Comparison with an appropriate reference standard (i.e. a data source other than notification data)? o Is the sensitivity of the reference standard described? Are cases verified? o Is the population base for notifications and supplementary data sources drawn from same catchment area? o Is there an adequate description of case ascertainment? o Are cases ascertained for the same time period in both datasets? • Are the data sources to be compared clearly identified and described/referenced? • What case definition is used? Is the same definition applied to all data sources? • Are the methods for linkage described? o Were the identifiers unique and available? o How complete was the link? (% linked) o How accurate was the link? (i.e. were individuals linked who were not supposed to be or vice versa) o Were duplicate cases identified and removed? • How precise are results? (confidence interval width) • Were any statistical measures of agreement used (did authors express level of agreement using a statistic)? • For capture-recapture methods: o If dependency between data sources is suspected, are ≥3 or more data sources used, along with log linear modelling methods, to account for this dependency?	Yes No Unclear N/A
	c) Is the research methodology free from bias? • Are there major sources of bias? • Can the study be reproduced?	Yes No Unclear N/A
	d) Are ethics procedures described? • Approval from ethics review board • Individual consent	Yes No Unclear N/A
	e) Can I be confident about findings? • Were sources of information quality assessed? • Any major methodological flaws?	Yes No Unclear N/A
Applicability	Can results be applied within the scope of public health? • Do the results of the study apply to the issue under consideration (i.e. are surveillance systems and supplementary data sources similar to Ontario?)?	Yes No Unclear N/A

^a^Reliability, section b was revised from the original tool based on studies from Doyle et al. (2002) and Pillaye and Clarke (2003), as well as the US CDC’s updated guidelines for evaluating public health surveillance systems [12, 28, 29]

### Statistical analysis

Reporting completeness was estimated as the number of diagnosed cases reported to public health divided by the total number of diagnosed cases ascertained through the reference standard. If the diagnosis was validated in the reference standard (e.g. ICD-9 coded hospital discharge records were reviewed to determine whether hepatitis A-coded visits met the surveillance case definition), the corrected denominator was used.

The pooled reporting completeness was derived by averaging study estimates, weighted by the inverse of their variance, and transformed using a double arcsine transformation [30]. The DerSimonian and Laird random-effects model was used to incorporate between-study variance and to pool the transformed proportions [31, 32]. Statistical heterogeneity was assessed by calculating Cochran’s Q statistic with a significance level of *P* < 0.10 and the I^2^ statistic [33], which reflects between-study heterogeneity. Values of above 75 % were used to indicate high heterogeneity [33]. All analyses were performed using MetaXL (http://www.epigear.com). Publication bias was assessed by visually examining Begg’s funnel plot and performing Egger’s regression asymmetry test using Stata, version 12.0 (StataCorp LP, College Station, Texas).

## Results

### Primary studies

**Fig. 1:**
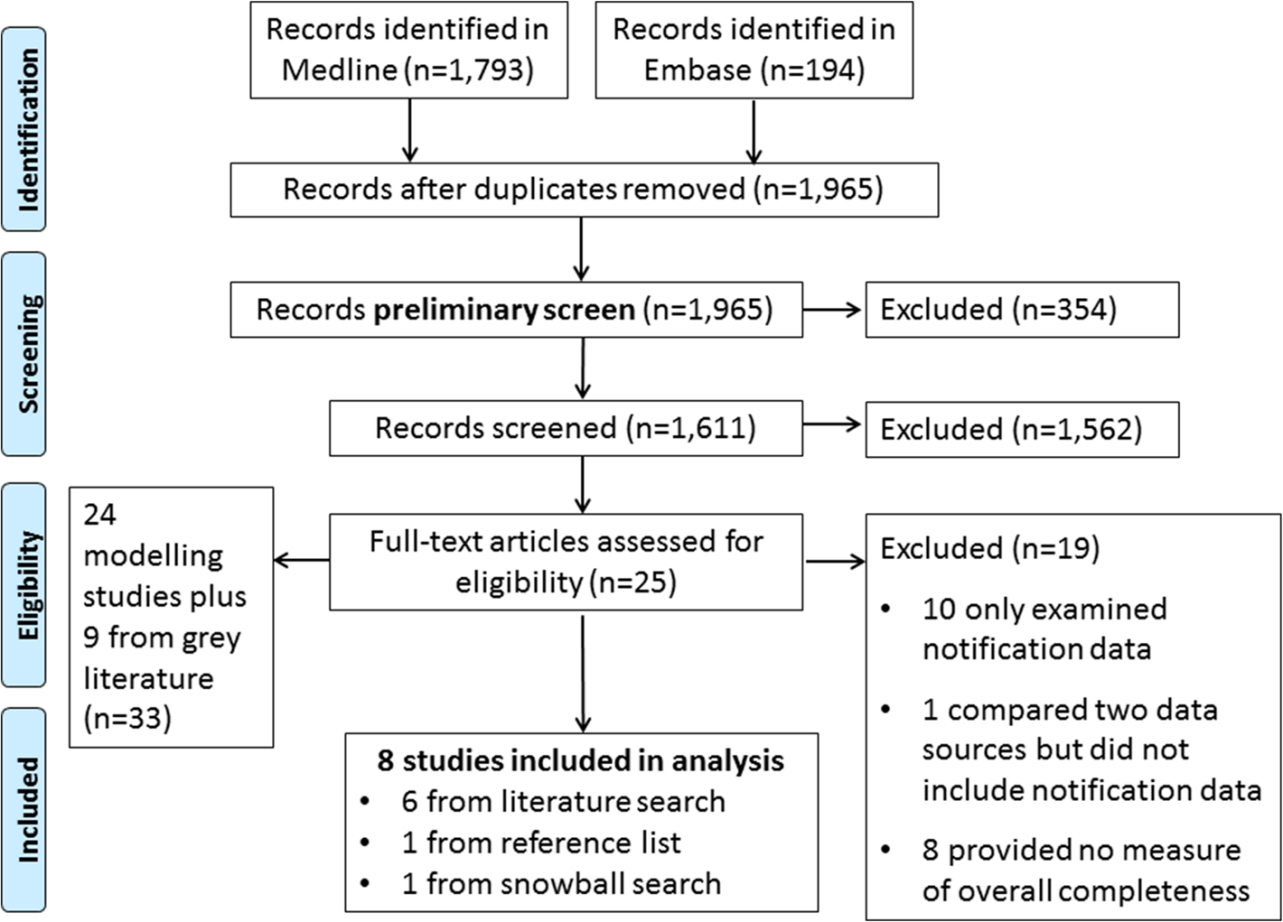
Flow chart of search strategy results and selection of papers

Overall, 1965 studies were retrieved from Medline and EMBASE, excluding duplicates (Fig. 1). After excluding 354 irrelevant studies in the preliminary review, 1611 studies were screened. Twenty-five studies were considered eligible for full-text review with good agreement between reviewers (kappa = 0.60, 95 % confidence interval (CI) = 0.45, 0.75) (see Additional file 2 for complete reference list). In total, eight studies were included; six from the search, one from the reference list of reviewed full-text articles, and one from the snowball search [34–41]. There was good agreement between reviewers at the final screen (kappa = 0.78, 95 % confidence interval (CI): 0.49, 1.00).

One relevant ECDC project, the ‘Current and Future Burden of Communicable Diseases in the European Union and EEA/EFTA countries’ (BCoDE) study, was identified in the grey literature search. Results from an unpublished literature scan conducted to aid in the development of multiplication factors to correct for under-ascertainment and underreporting of hepatitis A were shared by the study lead (written communication, Dr. Mirjam Kretzschmar). Four articles on underreporting were cited in this scan, only one of which met our inclusion/exclusion criteria and had already been retrieved and included in this review [40]. Annual viral hepatitis surveillance reports published on the US CDC website were also considered relevant as reports included adjustment for underreporting using a probabilistic model with factored probabilities of symptoms (I), referral to care and treatment (II), and rates of reporting to local and state department (III) [42]. The methodology is published and was retrieved in the original, peer-reviewed literature search [14]. Of the two model inputs for (III), the one study that met our inclusion/exclusion criteria had already been retrieved and included in this review [36].

### Modelling studies

In addition to the 25 primary studies reviewed in full-text, 24 modelling studies were reviewed (Additional file 2). An additional nine studies were reviewed from the BCoDE literature scan, for a total of 33 modelling studies (Additional file 2). No new primary studies on underreporting of hepatitis A were identified.

### Characteristics of included primary studies

Studies were published between 1998 and 2011, and conducted between 1995 and 2007 (Table 3). The majority (6/8) were from the US [34–37, 39, 41]; one study was from England [40] and another from New Zealand [38]. Hepatitis A was notifiable, meaning that it was legislated to be reported to public health, for all study settings during the study time periods. In most settings, there was a mandatory dual (physician and laboratory) reporting mechanism. Aside from one study that examined the proportion of positive test results identified within a managed care organization’s reporting system and another study that examined electronic medical records from a group practice [34, 36], all studies were population-based, meaning that cases could emerge from anywhere in the population and through any healthcare setting. Only one study measured underreporting in an outbreak context [40].

**Table 3:** Characteristics of eight included studies published between January 1997 and May 2015

Study Characteristics					Methods				Results	
Study and Year	Location	Time Period	Study Population	Notification to Public Health	Design	Referent Data of Diagnosed Cases	Diagnostic criteria	Consistent with surveillance criteria and possible effect	% Report	N
Backer HD et al. 2001 [34]	California, US	1997	Kaiser Permanente Northern California members	Mandatory dual reporting	Data Linkage	Laboratory Tests	Positive IgM HAV antibody	No; Under-estimate completeness	88.4 %	402
Boehmer TK et al. 2011 [35]	Colorado, US	2003–2005	Population-based	Mandatory dual reporting	Data Linkage	Inpatient hospital discharges and medical chart review	ICD-9-CM codes 070.0 and 070.1 with review using surveillance definition	Yes	67 %	6
Klompas M et al. 2008 [36]	Massachu-setts, US	June 2006–July 2007	Patients of a multi-specialty group practice of 35 clinics	Not described	Data Linkage	Electronic medical records with e-support for public health system (ESP)	ALT or AST >2 times upper normal limit, or ICD-9 code 782.4 for jaundice, and positive IgM HAV antibody	No; Over-estimate	25 %	4
Matin N et al. 2006 [40]	England (North East and East Midlands), UK	2002 and 2003	Population-based	Mandatory reporting by physicians; voluntary reporting by laboratories with good participation	CRC	1. Cases identified by local public health, 2. laboratory tests and 3. genotyping results	Not described	Unclear	81.7 % (outbreak a) and 27.8 % (outbreak b)	236 and 1107
Overhage JM et al. 2008 [39]	Indiana- polis, Indiana, US	First quarter of 2001	Population-based	Mandatory dual reporting	Data Linkage	Hospital infection-control databases (IC), and an electronic laboratory reporting (ELR) database	Not described; system scans test results labels for a match to CDC notifiable condition mapping tables	Unclear	4.0 % (IC, study a) and 97.3 % (ELR, study b)	150
Roels TH et al. 1998 [37]	Wisconsin, US	1995	Population-based	Mandatory dual reporting	Data Linkage/Compar-ison	Laboratory Tests	Positive IgM HAV antibody	No; Under-estimate	74 %	156
Sickbert-Bennett EE et al. 2011 [41]	North Carolina, US	1995–2006 (excl. 1998, 1999)	Population-based	Mandatory reporting by physicians; dual reporting starting 1998	Data Linkage	Inpatient hospital discharges and medical chart review	ICD-9-CM (code not specified) with review using surveillance definition	Yes	40.02 % corrected	67
Simmons G et al. 2002 [38]	Auckland, New Zealand	2000	Population-based	Mandatory reporting by physicians; some laboratories	Data Linkage	Laboratory Tests	Positive IgM HAV antibody	No; Under-estimate	65 %	54

*US* United States, *IgM* immunoglobulin M, *HAV* hepatitis A virus, *ICD* international classification of diseases, *ALT* alanine aminotransferase, *AST* asparatate aminotransferase, *UK* United Kingdom, *CRC* capture-recapture methods, *n/a* not available

### Risk of bias

There was good agreement between the two reviewers for bias assessment (kappa = 0.79, 95 % confidence interval (CI): 0.41, 1.00). Overall, two studies were assessed to have a low risk of bias, four studies a moderate risk, and two a high risk (Table 4). One of the most common study limitations was a failure to verify the diagnosis in the reference standard. For example, only two studies that used ICD-9 coded hospital discharge data to ascertain cases of hepatitis A verified the diagnosis though medical chart review [35, 41]. Study authors found that this process identified several false positives, which had they not been detected, would have led to an artificially low completeness of reporting estimate. For studies that linked diagnostic and public health data to estimate underreporting, the methods for matching and accuracy of the match were poorly described in most studies. Additionally, several studies did not report the case definitions or criteria used to ascertain cases and whether the definitions were consistent across data sources [34, 40]; although, it should be noted that a national surveillance case definition for hepatitis A did not exist in England at the time of the study conducted by Matin et al. [40] Reportable disease surveillance data varies by system design and implementation; despite this, most studies provided only a cursory description of the various data sources and population coverage of those sources and did not include relevant contextual information important to interpreting reporting completeness estimates.

**Table 4:** Risk of bias assessment using Public Health Ontario’s meta-tool for quality assessment [25]

Item	Criteria	Backer 2001 [34]	Boehmer 2011 [35]	Klompas 2008 [36]	Matin 2006 [40]	Overhage 2008 [39]	Roels 1998 [37]	Sickbert-Bennett 2011 [41]	Simmons 2002 [38]
Relevancy	Topic(s) relevant	Yes	Yes	Yes	Yes	Yes	Yes	Yes	Yes
Validity	Presented objectively	Unclear	Unclear	Unclear	Unclear	Yes	Yes	Yes	Yes
	Conclusions justified	Yes	Yes	Yes	Yes	Yes	Yes	Yes	Yes
Reliability	Clear methods	Yes	Yes	Yes	Yes	Unclear	Unclear	Yes	Unclear
	Appropriate methods	Unclear	Yes	Yes	Yes	Unclear	Unclear	Yes	Yes
	Free from bias	No	Yes	Unclear	No	Unclear	Unclear	No	Unclear
	Ethics described	Unclear	Unclear	Unclear	Unclear	No	Unclear	Yes	Yes
	Confident in findings	Unclear	Yes	Unclear	Unclear	No	No	Unclear	No
Applicability	Applicable	Unclear	Yes	Yes	Unclear	Unclear	Yes	Yes	Yes
Overall Risk		High	Low	Moderate	Moderate	High	Moderate	Low	Moderate

### Data synthesis

**Fig. 2:**
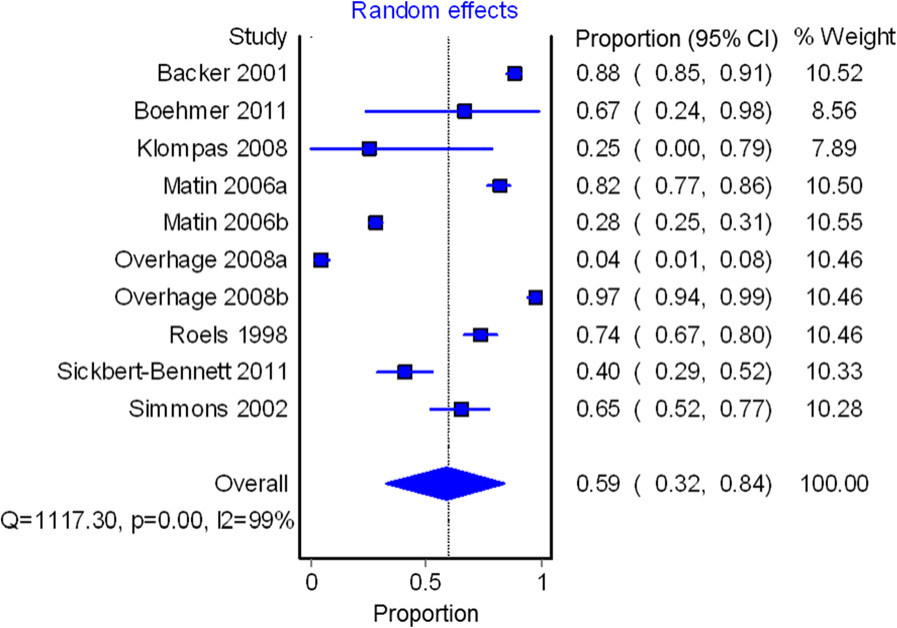
Pooled proportion of hepatitis A reporting completeness to public health in eight studies. *CI* confidence interval

In all included studies, hepatitis A reporting was found to be incomplete. The proportion of hepatitis A cases reported to public health ranged from 4 to 97 % (pooled proportion = 59 %, 95 % CI = 32 %, 84 %) (Table 3 and Fig. 2). High heterogeneity was observed (*Q* = 1117.30, *P* < 0.001; I^2^ = 99 %). Reporting completeness estimates were not stratified by age, sex or risk status in any of the included studies (data not shown).

**Fig. 3:**
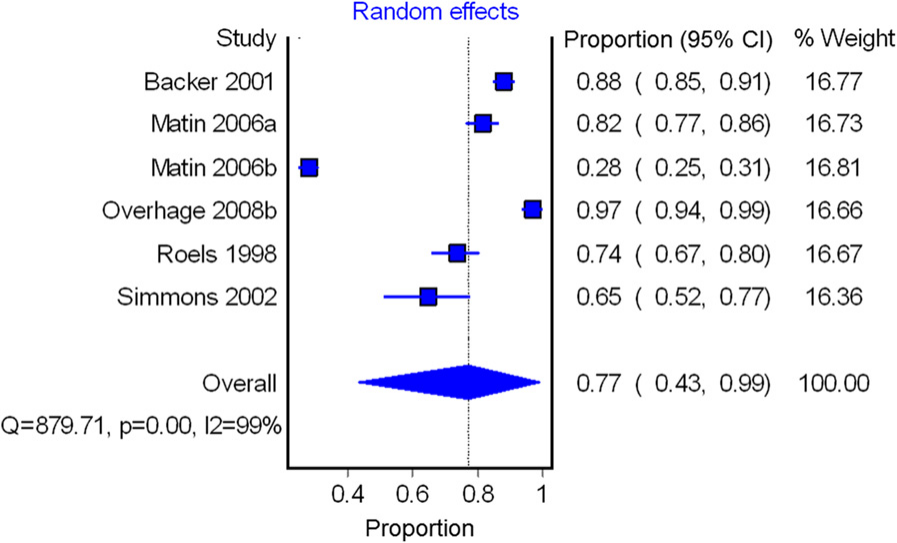
Pooled hepatitis A reporting completeness in studies with laboratory testing data as the reference standard. *CI* confidence interval

The majority of studies (7/8) linked public health notification data with an independently retrieved data source of diagnosed cases to measure underreporting (Table 3) [34–39, 41]. The most commonly used reference standard was positive IgM anti-HAV test results from laboratories [34, 37–39]; two studies used inpatient hospital discharge data validated through medical record review to assess underreporting [35, 41], while another used electronic medical records [36]. Only one study used capture-recapture methods applied to two hepatitis A outbreaks to measure underreporting [40]. The heterogeneity in reporting completeness may be explained in part by the differing standards used. In a post-hoc subgroup analysis, studies which assessed completeness by comparing public health data with laboratory testing data found that a higher proportion of cases were reported to public health (pooled proportion = 77 %, 95 % CI = 43 %, 99 %); however, significant residual heterogeneity remained (Fig. 3).

With the exception of one study [37], no studies reported which anti-HAV IgM test was used, the sensitivity and specificity of the test, and whether there were any changes to laboratory testing procedures over the study time period, making it difficult to assess whether the heterogeneity could be explained by differences in testing practices/methods. The variation could also be explained by differences in how (or the mechanism by which) cases were reported to public health (e.g. automated versus manual reporting methods, electronic versus paper, staff resource level, etc.) and legislation across and within study settings. For example, higher estimates of reporting completeness were observed in the two studies with automated laboratory reporting (97 and 88 %) [34, 39] relative to the other two studies that relied on manual reporting methods (74 and 65 %) [37, 38]. The latter also cited specific challenges with reporting including poor information exchange with private laboratories [37], and lack of routine reporting by selected laboratories [38]. If data from these four studies were pooled, cases detected in automated reporting systems would have had 3.92 times the odds of being reported to public health compared to cases detected in manual reporting systems. Other studies that similarly relied on manual, passive reporting but in different settings (by infection control practitioners [39] and primary care providers [36]) also found lower proportions of complete reporting (4 and 25 %). In addition to between study variation, Sickbert-Bennett et al. (2011) noted heterogeneity in reporting mechanisms *within* one health region, finding higher reporting completeness in hospitals with dedicated staff (i.e. public health epidemiologists or infection control practitioners) responsible for disease reporting [41].

**Fig. 4:**
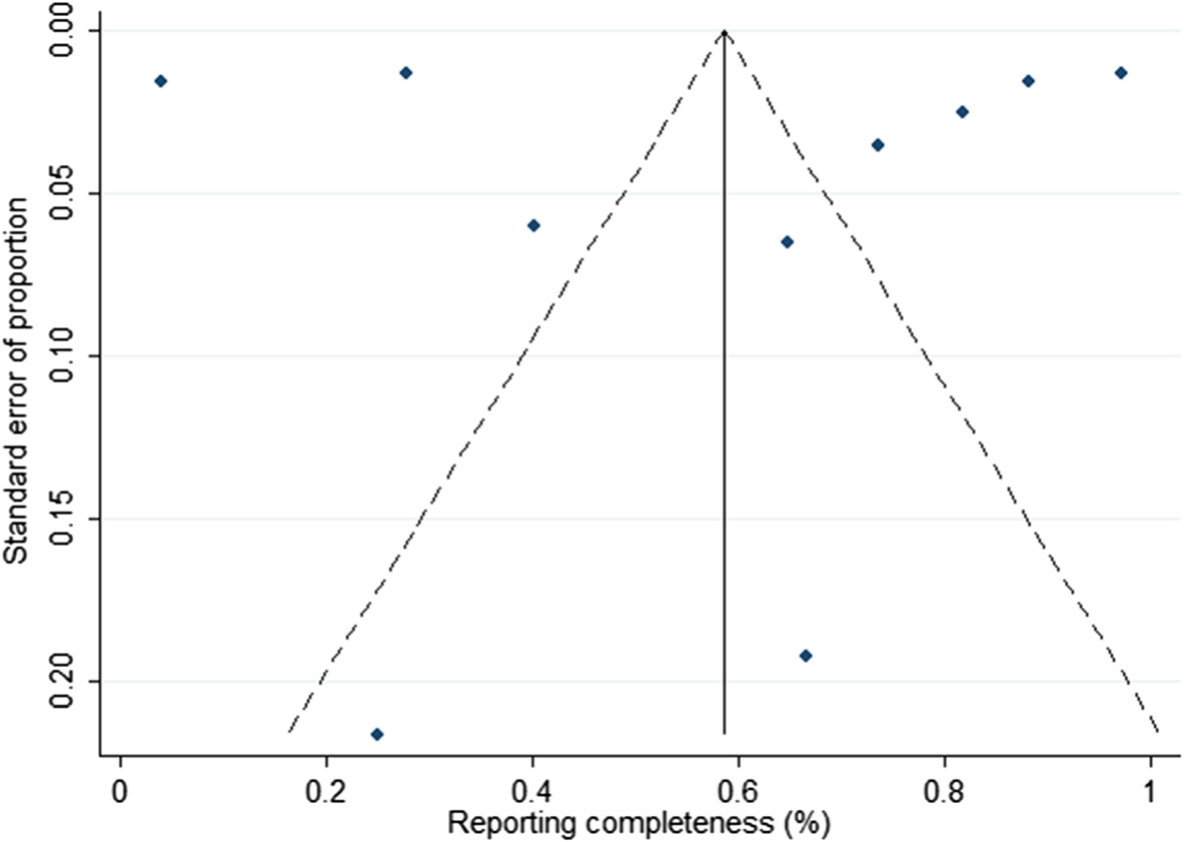
Funnel plot for assessing publication bias of hepatitis A reporting completeness in non-endemic countries

There was no evidence of publication bias based on funnel plot symmetry between the proportion of reporting completeness and the standard error of the proportion (Fig. 4) or from Egger’s test (*P* = 0.977).

## Discussion

The body of literature on the sensitivity of passive surveillance systems to capture diagnosed hepatitis A cases reveals that hepatitis A is underreported in non-endemic countries. The majority of studies were conducted in the US; despite this, there was substantial variability in reporting completeness (range: 4 to 97 %). Previous systematic reviews on completeness of disease notification for all reportable diseases in the US and UK similarly found variability in estimates depending on the disease, ranging from 9 to 99 % and 3 to 95 %, respectively [12, 20]. These studies, however, covered all notifiable diseases and found that reporting completeness was strongly correlated to the disease itself and not to study characteristics such as study location, time period, study design, and study size. Consequently, heterogeneity in estimates was attributed to the wide number of diseases being evaluated.

We found that the disparate reference standards (data sources of diagnosed cases) used by the studies in this review contributed in part to the observed variation in reporting completeness; however, substantial heterogeneity remained among studies which evaluated reporting completeness through comparison with laboratory testing data. Reporting completeness was highest where laboratory reporting to public health was automated relative to manual reporting methods and in settings where dedicated staff were responsible for disease reporting. These findings provide specific direction as to how surveillance systems can be improved to minimize underreporting.

Differences in important contextual factors, namely disease incidence and vaccination policy, in US states may have also influenced laboratory and physician reporting completeness. Unlike New Zealand and England vaccine recommendations which target high risk groups, HAV vaccination recommendations in the US have employed a phased approach, starting first with high risk groups, then moving in 1999 to target 17 states with reported rates above the national average [23] and in 2006 to all children over 1 year of age [5]. California and Colorado were both included in the targeted approach in 1999; it is possible that the improved reporting completeness relative to the studies from other settings was a result of heightened vigilance of reporting in this specific context [34, 35].

Other contributing factors to the observed heterogeneity could include varying study time periods, quality, sample size and/or legislative requirements for notification; although, no clear trend was observed across these factors. There may be additional features unique to each surveillance system that influence reporting completeness (e.g. regular auditing or other quality improvement activities, etc.). These features are challenging to elucidate, however, as many studies only provided a cursory description of their surveillance system, despite recommendations from the US CDC’s Guidelines Working Group for Evaluating Public Health Surveillance Systems to report on the context, operations, and system components, including the level of integration with other systems, when conducting an evaluation [23]. Efforts to contact study authors to obtain this information were either unsuccessful or impeded by recall issues. As this information is vital to identify those unique features that improve or impede system sensitivity, and can be used to improve the quality of systems everywhere, we recommend that future evaluation studies prioritize the inclusion of this information.

Methodological limitations identified in included studies provide further insight into how future studies evaluating the sensitivity of surveillance systems can be improved (recommendations summarized in Table 5). Several studies did not describe how cases were diagnosed in the reference standard and many did not take steps to validate the diagnosis in any way. For studies using positive IgM anti-HAV laboratory test results as the reference standard, false positive test results or repeat testing may have overestimated the denominator and underestimated the proportion of cases reported to public health, although, the specificity of IgM anti-HAV testing has been shown to be ≥99 % [43]. By contrast, lack of clinical information in an electronic medical record may have resulted in false negatives, which would have overestimated reporting completeness [36]. Application of a different case definition in the reference standard from that used for surveillance may have also led to disease misclassification. For example, using positive IgM anti-HAV laboratory test results alone to ascertain cases in laboratory data without consideration of the presence of clinically compatible symptoms, a component of the surveillance case definition, may have underestimated reporting completeness. Similarly, studies have demonstrated that using only ICD-9 hepatitis A discharge diagnosis codes, without verification by medical chart review, overestimates cases as defined by the surveillance case definition [35, 41]. Lastly, methods used to match records in the diagnostic and public health datasets, such as deterministic or probabilistic linkage, and the accuracy of the match was often not described; record linkage errors may have similarly underestimated reporting completeness or resulted in other errors. Given that surveillance reports are typically brief in nature, it is unclear whether these concerns reflect true methodological issues, or issues related to reporting quality.

**Table 5:** Summary of recommendations for future studies evaluating completeness of surveillance by linking public health surveillance data with a reference standard

1. Report on the context, operations, and system components of the public health surveillance system being evaluated, including the level of integration with other systems.	
2. Provide the surveillance case definition (if available), and describe how cases were diagnosed in the reference standard. If possible, apply the surveillance case definition to the reference standard to avoid misclassification in ascertaining cases.	
3. Validate the diagnosis of cases in the reference standard if possible, particularly if using inpatient hospital discharges or electronic medical records as reference standards.	
4. Describe methods used to match or link records in the diagnostic and public health datasets, such as deterministic or probabilistic linkage, and the accuracy of the match.	

Strengths of this review include a comprehensive search strategy which included two key scientific literature databases and relevant grey literature sources identified by experts in hepatitis A and public health surveillance. Search methods were validated using modelling studies and communication with public health professionals at two core knowledge-generating public health organizations. Additionally, adaptation of the critical appraisal tool better facilitated the identification of fundamental issues affecting each included study’s internal validity.

The scope of the study was restricted to focus on underreporting and not under-ascertainment as reporting practices are amenable to quality improvement, while issues related to under-ascertainment are less so, particularly when cases are asymptomatic, mild or have self-limiting infections that do not require medical care. Nonetheless, under-ascertainment remains an important contributor to the sensitivity of passive surveillance systems and can have substantial effects on disease control and prevention. Improving health literacy (knowledge of the severity or duration of an illness, and when to seek care), and removing administrative, financial and cultural barriers to healthcare may improve case ascertainment [19]. From a methodological perspective, the degree to which under-ascertainment occurs for a particular disease in a community can be estimated through the use of a wide range of active case finding methods, including serial serosurveys, as described by Gibbons et al. (2014) [19]. These estimates can then, in turn, be used to calibrate surveillance data.

## Conclusions

Given that complete and timely reporting of notifiable diseases is important to prevent outbreaks and person-to-person transmission, efforts should be undertaken to improve and evaluate surveillance on an ongoing basis. Automating case reporting to public health and/or providing dedicated staffing infrastructure with clear staff reporting responsibilities can improve reporting completeness. To identify additional modifiable features of surveillance systems and provide specific direction on how to improve system sensitivity and utility, future studies should describe the context, operations, and components of their system when conducting an evaluation, as well as critically reflect on how these features contributed to low or high sensitivity. Lastly, priority should be given to improving the quality of risk factor data recorded in surveillance systems to enable estimation of reporting completeness across high risk groups; these data are essential to examine equity issues, inform allocation of public health resources including control measures, and evaluate the effectiveness of public health interventions including vaccine recommendations.

## Abbreviations

HAV, hepatitis A virus; IgM, immunoglobulin M; PRISMA, Preferred Reporting Items for Systematic Reviews and Meta-Analyses; WHO, World Health Organization; US CDC, United States Centers for Disease Control and Prevention; PHAC, Public Health Agency of Canada; ECDC, European Centre for Disease Prevention and Control; ICD, International Classification of Diseases.

## Additional files

Additional file 1: Preferred Reporting Items for Systematic Reviews and Meta-Analyses (PRISMA) statement checklist. (DOCX 29 kb) Additional file 2: Reference list of all articles reviewed in full-text. (DOCX 26 kb)
