# Supplementary material for: Underreporting of hepatitis A in non-endemic countries: a systematic review and meta-analysis
Source: BMC Infect Dis. 2016 Jun 13;16:281. doi: 10.1186/s12879-016-1636-6 (PMC4906888; doi:10.1186/s12879-016-1636-6)
Supplement: Additional file 2: — Reference list of all articles reviewed in full-text. (DOCX 26 kb) [file 12879_2016_1636_MOESM2_ESM.docx]

**Additional File 2. Reference list of all articles reviewed in full-text.**

1. Allen CJ, Ferson MJ: Notification of infectious diseases by general practitioners: a quantitative and qualitative study. Med J Aust 2000, 172(7):325-328.

2. Allen-Dicker J, Klompas M: Comparison of electronic laboratory reports, administrative claims, and electronic health record data for acute viral hepatitis surveillance. Journal of Public Health Management & Practice 2012, 18(3):209-214.

3. Backer HD, Bissell SR, Vugia DJ: Disease reporting from an automated laboratory-based reporting system to a state health department via local county health departments. Public Health Rep 2001, 116(3):257-265.

4. Boehmer TK, Patnaik JL, Burnite SJ, Ghosh TS, Gershman K, Vogt RL: Use of hospital discharge data to evaluate notifiable disease reporting to Colorado's Electronic Disease Reporting System. Public Health Rep 2011, 126(1):100-106.

5. Centers for Disease Control and Prevention (CDC): Potential effects of electronic laboratory reporting on improving timeliness of infectious disease notification--Florida, 2002-2006. MMWR - Morbidity & Mortality Weekly Report 2008, 57(49):1325-1328.

6. Centers for Disease Control and Prevention (CDC): Automated detection and reporting of notifiable diseases using electronic medical records versus passive surveillance--Massachusetts, June 2006-July 2007. MMWR - Morbidity & Mortality Weekly Report 2008, 57(14):373-376.

7. Iqbal K, Klevens RM, Jiles R: Comparison of acute viral hepatitis data quality using two methodologies, 2005-2007. Public Health Rep 2012, 127(6):591-597.

8. Jajosky RA, Groseclose SL: Evaluation of reporting timeliness of public health surveillance systems for infectious diseases. BMC Public Health 2004, 4:29.

9. Leung TW, Vrbova L: Descriptive analysis of endemic and travel hepatitis A cases in Ontario, 1998 to 2004. Can Commun Dis Rep 2006, 32(24):287-296.

10. Markov PV, Ahmed SN, Crowcroft NS: Hepatitis A virus infection control: an audit of practice in England and Wales prior to guidelines being published. Communicable Disease & Public Health 2003, 6(1):30-33.

11. Matin N, Grant A, Granerod J, Crowcroft N: Hepatitis A surveillance in England--how many cases are not reported and does it really matter?. Epidemiology & Infection 2006, 134(6):1299-1302.

12. Moore KM, Reddy V, Kapell D, Balter S: Impact of electronic laboratory reporting on hepatitis A surveillance in New York City. Journal of Public Health Management & Practice 2008, 14(5):437-441.

13. Reijn E, Swaan CM, Kretzschmar ME, van Steenbergen JE: Analysis of timeliness of infectious disease reporting in the Netherlands. BMC Public Health 2011, 11:409.

14. Roels TH, Christl M, Kazmierczak JJ, MacKenzie WR, Davis JP: Hepatitis A infections in Wisconsin: trends in incidence and factors affecting surveillance, 1986-1995. WMJ 1998, 97(5):32-38.

15. Simmons G, Whittaker R, Boyle K, Morris AJ, Upton A, Calder L: Could laboratory-based notification improve the control of foodborne illness in New Zealand?. N Z Med J 2002, 115(1154):237-240.

16. Smallman-Raynor M, Cliff AD, Haggett P, Stroup DF, Williamson GD: Spatial and temporal patterns in final amendments to provisional disease counts. Journal of Public Health Management & Practice 1999, 5(3):68-83.

17. Ward M, Borgen K, Mazick A, Muehlen M: Hepatitis A vaccination policy for travellers to Egypt in eight European countries, 2004. Euro Surveill 2006, 11(1):37-39.

18. Itani T, Jacobsen KH, Nguyen T, Wiktor SZ: A new method for imputing country-level estimates of hepatitis A virus endemicity levels in the Eastern Mediterranean region. Vaccine 2014, 32(46):6067-6074.

19. Stachel AG, Waechter H, Bornschlegel K, Reddy V, Hanson H, Wen T, et al: Reassessing provider reporting in the age of electronic surveillance. J Public Health Manag Pract 2014, 20(2):240-245.

20. Petrignani M, Verhoef L, Vennema H, van Hunen R, Baas D, van Steenbergen JE, et al: Underdiagnosis of foodborne hepatitis A, The Netherlands, 2008-2010(1.). Emerg Infect Dis 2014, 20(4):596-602.

21. Montano-Remacha C, Ricotta L, Alfonsi V, Bella A, Tosti M, Ciccaglione A, et al: Hepatitis A outbreak in Italy, 2013: a matched case-control study. Euro Surveill 2014, 19(37):20906.

22. La Rosa G, Libera SD, Iaconelli M, Ciccaglione AR, Bruni R, Taffon S, et al: Surveillance of hepatitis A virus in urban sewages and comparison with cases notified in the course of an outbreak, Italy 2013. BMC Infect Dis 2014, 14:419-2334-14-419.

23. FitzSimons D, McMahon B, Hendrickx G, Vorsters A, Van Damme P: Burden and prevention of viral hepatitis in the Arctic region, Copenhagen, Denmark, 22-23 March 2012. Int J Circumpolar Health 2013, 72: doi: 10.3402/ijch.v72i0.21163.

24. Nordic Outbreak Investigation Team C: Joint analysis by the Nordic countries of a hepatitis A outbreak, October 2012 to June 2013: frozen strawberries suspected. Euro Surveill 2013, 18(27):20520.

25. Rizzo C, Alfonsi V, Bruni R, Busani L, Ciccaglione A, De Medici D, et al: Ongoing outbreak of hepatitis A in Italy: preliminary report as of 31 May 2013. Euro Surveill 2013, 18(27):20518.

26. Overhage JM, Grannis S, McDonald CJ: A comparison of the completeness and timeliness of automated electronic laboratory reporting and spontaneous reporting of notifiable conditions. Am J Public Health 2008, 98(2):344-350.

27. Sickbert-Bennett EE, Weber DJ, Poole C, MacDonald PD, Maillard JM: Completeness of communicable disease reporting, North Carolina, USA, 1995-1997 and 2000-2006. Emerg Infect Dis 2011, 17(1):23-29.

28. Ajelli M, Iannelli M, Manfredi P, Ciofi degli Atti ML: Basic mathematical models for the temporal dynamics of HAV in medium-endemicity Italian areas. Vaccine 2008, 26(13):1697-1707.

29. Ajelli M, Merler S: An individual-based model of hepatitis A transmission. J Theor Biol 2009, 259(3):478-488.

30. Ajelli M, Fumanelli L, Manfredi P, Merler S: Spatiotemporal dynamics of viral hepatitis A in Italy. Theor Popul Biol 2011, 79(1-2):1-11.

31. Amariei R, Willms AR, Bauch CT: The United States and Canada as a coupled epidemiological system: an example from hepatitis A. BMC Infectious Diseases 2008, 8:23.

32. Armstrong GL, Bell BP: Hepatitis A virus infections in the United States: model-based estimates and implications for childhood immunization. Pediatrics 2002, 109(5):839-845.

33. Bauch CT, Rao AS, Pham BZ, Krahn M, Gilca V, Duval B, et al: A dynamic model for assessing universal Hepatitis A vaccination in Canada. Vaccine 2007, 25(10):1719-1726.

34. Bauch CT, Anonychuk AM, Pham BZ, Gilca V, Duval B, Krahn MD: Cost-utility of universal hepatitis A vaccination in Canada. Vaccine 2007, 25(51):8536-8548.

35. Held L, Hofmann M, Hohle M, Schmid V: A two-component model for counts of infectious diseases. Biostatistics 2006, 7(3):422-437.

36. Shkedy Z, Aerts M, Molenberghs G, Beutels P, Van Damme P: Modelling age-dependent force of infection from prevalence data using fractional polynomials. Stat Med 2006, 25(9):1577-1591.

37. Van Effelterre TP, Zink TK, Hoet BJ, Hausdorff WP, Rosenthal P: A mathematical model of hepatitis a transmission in the United States indicates value of universal childhood immunization. Clinical Infectious Diseases 2006, 43(2):158-164.

38. Watkins RE, Eagleson S, Veenendaal B, Wright G, Plant AJ: Disease surveillance using a hidden Markov model. BMC Medical Informatics & Decision Making 2009, 9:39.

39. Amin J, Gilbert GL, Escott RG, Heath TC, Burgess MA: Hepatitis A epidemiology in Australia: national seroprevalence and notifications. Med J Aust 2001, 174(7):338-341.

40. Anonychuk AM, Tricco AC, Bauch CT, Pham B, Gilca V, Duval B, et al: Cost-effectiveness analyses of hepatitis A vaccine: a systematic review to explore the effect of methodological quality on the economic attractiveness of vaccination strategies. Pharmacoeconomics 2008, 26(1):17-32.

41. Ansaldi F, Bruzzone B, Rota MC, Bella A, Ciofi degli Atti M, Durando P, et al: Hepatitis A incidence and hospital-based seroprevalence in Italy: a nation-wide study. Eur J Epidemiol 2008, 23(1):45-53.

42. Berge JJ, Drennan DP, Jacobs RJ, Jakins A, Meyerhoff AS, Stubblefield W, et al: The cost of hepatitis A infections in American adolescents and adults in 1997. Hepatology 2000, 31(2):469-473.

43. Gervelmeyer A, Nielsen MS, Frey LC, Sckerl H, Damberg E, Molbak K: An outbreak of hepatitis A among children and adults in Denmark, August 2002 to February 2003. Epidemiology & Infection 2006, 134(3):485-491.

44. Hesketh LM, Rowlatt JD, Gay NJ, Morgan-Capner P, Miller E: Childhood infection with hepatitis A and B viruses in England and Wales. CDR Review 1997, 7(4):R60-3.

45. Oviedo M, Munoz MP, Dominguez A, Carmona G: Estimated incidence of hepatitis A virus infection in Catalonia. Ann Epidemiol 2006, 16(11):812-819.

46. Oviedo M, Pilar Munoz M, Dominguez A, Borras E, Carmona G: A statistical model to estimate the impact of a hepatitis A vaccination programme. Vaccine 2008, 26(48):6157-6164.

47. Pham B, Chen MH, Tricco AC, Anonychuk A, Krahn M, Bauch CT: Use of a catalytic model to estimate hepatitis A incidence in a low-endemicity country: implications for modeling immunization policies. Medical Decision Making 2012, 32(1):167-175.

48. Srinivasa Rao AS, Chen MH, Pham BZ, Tricco AC, Gilca V, Duval B, et al: Cohort effects in dynamic models and their impact on vaccination programmes: an example from hepatitis A. BMC Infectious Diseases 2006, 6:174.

49. Teitelbaum P: An estimate of the incidence of hepatitis A in unimmunized Canadian travelers to developing countries. Journal of Travel Medicine 2004, 11(2):102-106.

50. Klevens RM, Liu S, Roberts H, Jiles RB, Holmberg SD: Estimating acute viral hepatitis infections from nationally reported cases. Am J Public Health 2014, 104(3):482-487.

51. Bonacic Marinovic A, Swaan C, van Steenbergen J, Kretzschmar M: Quantifying reporting timeliness to improve outbreak control. Emerg Infect Dis 2015, 21(2):209-216.

52. Havelaar AH, Haagsma JA, Mangen MJ, Kemmeren JM, Verhoef LP, Vijgen SM, et al: Disease burden of foodborne pathogens in the Netherlands, 2009. Int J Food Microbiol 2012, 156(3):231-238.

53. Szucs T: Cost-effectiveness of hepatitis A and B vaccination programme in Germany. Vaccine 2000, 18(Suppl 1):S86-9.

54. Jacobs RJ, Margolis HS, Coleman PJ: The cost-effectiveness of adolescent hepatitis A vaccination in states with the highest disease rates. Arch Pediatr Adolesc Med 2000, 154(8):763-770.

55. O'Connor JB, Imperiale TF, Singer ME: Cost-effectiveness analysis of hepatitis A vaccination strategies for adults. Hepatology 1999, 30(4):1077-1081.

56. Mead PS, Slutsker L, Dietz V, McCaig LF, Bresee JS, et al: Food-related illness and death in the United States. Emerg Infect Dis 1999, 5(5):607-625.

57. Scallan E, Hoekstra RM, Angulo FJ, Tauxe RV, Widdowson MA, Roy SL, et al: Foodborne illness acquired in the United States--major pathogens. Emerg Infect Dis 2011, 17(1):7-15.

58. Greengold B, Nyamathi A, Kominski G, Wiley D, Lewis MA, Hodge F, et al: Cost-effectiveness analysis of behavioral interventions to improve vaccination compliance in homeless adults. Vaccine 2009, 27(5):718-725.

59. Das A: An economic analysis of different strategies of immunization against hepatitis A virus in developed countries. Hepatology 1999, 29(2):548-552.

60. Beutels P, Luyten J, LeJeune O, Hens N, Bilcke J: Evaluation of universal and targeted hepatitis A vaccination programs in Belgium. *KCE Reports 98B* 2008.
